# The Role and Validity of Diagnostic Biomarkers in Late-Onset Neonatal Sepsis

**DOI:** 10.7759/cureus.17065

**Published:** 2021-08-10

**Authors:** Patience Mwesigye, Fizza Rizwan, Niazy Alassaf, Rizwan Khan

**Affiliations:** 1 Internal Medicine, University of Limerick, Limerick, IRL; 2 Internal Medicine, Medical University of Sofia, Sofia, BGR; 3 Neonatology, University Maternity Hospital, Limerick, Limerick, IRL

**Keywords:** neonatal, sepsis, biomarkers, late-onset, diagnosis

## Abstract

Sepsis remains a leading cause of mortality in the neonatal population, and currently, there is still no consensus on an accurate biomarker that can aid prompt diagnosis. This review focuses on studies investigating biomarkers for late-onset neonatal sepsis specifically. We discuss the current evidence for traditionally used biomarkers and present recent developments on more novel markers.

Suitable articles were selected from PubMed, Embase, Medline, Cochrane Handbook of Systematic Reviews, and ScienceDirect. Inclusion criteria were studies published from 2010 to 2020. Exclusion criteria were animal model-based studies. Keywords in search strategy were late-onset neonatal sepsis + biomarkers + diagnosis.

Evidence is growing increasingly weak for commonly studied biomarkers such as C-reactive protein (CRP) and procalcitonin (PCT). Levels of markers such as Serum Amyloid A and Neutrophil CD64 increase more rapidly post-onset of infection compared to CRP. Moreover, this review found that the more novel biomarkers discussed such as presepsin and endocan may show superior and more promising potential as diagnostic markers. However, larger studies over multicenters are deemed essential to ascertain the ideal biomarker.

## Introduction and background

Introduction

Neonatal sepsis is a clinical syndrome that occurs due to bacterial bloodstream infections and leads to abnormalities in systemic circulation [1]. In newborn infants, these infections occur during the first 28 days of life and are classified into early-onset sepsis (EOS) and late-onset sepsis (LOS) [1]. While EOS is due to ascending infections from the maternal genital tract, LOS occurs due to postnatal nosocomial environment or within the community, usually later than 72 hours after birth [2]. 

To this day, LOS remains a leading cause of morbidity and mortality within neonates due to its associated myriad of complications such as neurodevelopmental impairment, bronchopulmonary dysplasia, ductus arteriosus, and necrotizing enterocolitis [1]. However, its diagnosis remains challenging due to vague clinical symptoms that result in delayed assessment and treatment [1]. These vague clinical signs usually overlap with common un-infectious conditions such as transitional tachypnea of the newborn and apnea of prematurity [3]. Consequently, this leads to unnecessary, prolonged, and/or failure of early and lifesaving diagnosis. Additionally, unnecessary antimicrobial treatments lead to increased drug resistance organisms, another area of worldwide concern [2]. 

Traditionally, a blood culture is the gold standard diagnosis for sepsis in neonates [2]. However, this method has several challenges including increased incidence of false-negative results and long waiting times for results [2]. Over the years, diagnostic biomarkers have become an area of interest in the study of neonatal sepsis. The ideal biomarker should have a high degree of sensitivity and specificity so as to avoid missing sepsis cases and exposing infants to unnecessary treatments [3]. It should also be able to easily measure and indicate prognostic information [3].

A few biomarkers have been investigated in an attempt to come up with a reliable and accurate diagnostic biomarker for neonatal sepsis, but those of LOS remain understudied. Most studies have been focused on EOS, which correlates to a general reduction in EOS incidents worldwide [3]. The incidence of LOS in very low birth weight infants is approximately 21%, a value 10 times higher than the incidence of EOS [4]. 

To date, very few studies have investigated biomarkers solely in the context of LOS. This, therefore, indicates the necessity of identifying these optimal markers and evaluating their clinical role.

The aim of this review is to discuss and highlight recent work on the role of biomarkers associated specifically to LOSlate-onset neonatal sepsis (LOS).

## Review

Methods

All studies in the review were selected using these databases, none were hand-selected. Studies relating to LOSlate-onset neonatal sepsis and biomarkers were selected. The most recent search was performed on June 14, 2020.

Articles were searched using the following databases: PubMed, Embase, Medline, Cochrane Handbook of Systematic Reviews, and ScienceDirect. Search terms used were: “‘Late-onset sepsis”’ + “‘neonates’” + “‘biomarkers’” + “‘diagnostic’”. 

(1) Inclusion criteria were studies performed with the above terminologies, ranging from 2010 to 2020. 

(2) Exclusion criteria were studies performed in 2009 or prior, studies performed on animal subjects, and studies focusing on overall neonatal sepsis, i.e., both early-onset and late-onset neonatal sepsis.

Following the application of this criteria, a total of 41 articles were found. Out of these, 30 were excluded as they did not meet the inclusion criteria. The reference lists of the 11 studies that were left were then further investigated, yielding eight more potential articles for review. Three more articles were then found to meet the inclusion criteria. Overall, 14 papers were selected for review. 

The LOS biomarkers identified within the papers included C-reactive protein (CRP), procalcitonin (PCT), serum amyloid A (SAA), presepsin, and neutrophil CD64. Therefore emphasis was directed on these biomarkers.

Results

1. Procalcitonin

Procalcitonin (PCT) is a pro-peptide of calcitonin. It is produced by the parafollicular cells of the thyroid gland and consists of 116 amino acids. Unlike calcitonin, PCT lacks hormonal activity but has been linked to sepsis as its levels rise rapidly in patients with bacterial infections [4].

PCT levels normally rise two to four days after birth, but they rapidly increase in states of infection and can be detected six hours post-infection, peaking at 18-24 hours and remain elevated for up to 48 hours [5]. PCT is not only associated with the onset of the infection but also its severity as its levels decrease rapidly after antimicrobial therapy is administered [5]. PCT indicates a specificity for sepsis as its levels are almost undetectable (0.033 ng/ml) in healthy individuals [4].

Bustos et al. did a prospective observational study on a cohort of 53 preterm-term/low birth weight newborns who had clinical suspicion of LOS [4]. These neonates were divided into confirmed sepsis versus clinical sepsis groups. Compared to the clinical sepsis cohort, plasma PCT levels were found to be significantly higher within the confirmed sepsis group.

The results of this study showed that PCT had a sensitivity of 88%, specificity of 71.4%, and a negative predictive value of 87%. Comparatively, it was shown that CRP had a sensitivity of 12% and specificity of 100% [4].

2. C-Reactive Protein

C-reactive protein (CRP) is an acute-phase protein released by the liver under stimulation by pro-inflammatory cytokines. Traditionally, serum CRP is measured as part of the initial investigations in sepsis diagnosis and is used as a rapid test to guide management in infants with suspected LOS [6].

Beltempo et al. conducted a five-year retrospective cohort study involving 416 eligible infants [7]. They found that combining both complete blood count (CBC) and CRP offered the highest sensitivity for LOS diagnosis. This was statistically superior to the individual potential of either marker. While CRP had a higher individual sensitivity of 89% compared to CBC at 59%, it was shown that overall, combining CRP and CBC had the highest sensitivity of 88% and a negative predictive value of 93% [7]. This emphasized that CRP shows diagnostic potential only when used in combination with other markers. 

In a meta-analysis by Brown et al., the combined analysis indicated that a positive CRP test correctly identified sepsis infants about six times out of 10 [6]. When the data was applied to a hypothetical cohort of 1,000000 newborn infants with suspected late-onset infection, it was estimated that about 152 cases of infection would be missed (i.e., false negatives), and 156 non-infected infants would be wrongly diagnosed (i.e., false positive) [6]. Therefore, serum CRP levels are unlikely to be considered sufficiently accurate to aid early diagnosis or improve the accuracy of treatment.

3. Serum Amyloid A and Apolipoprotein C-II

Serum Amyloid A (SAA) are proteins used as markers for acute and chronic inflammation [8]. They belong to a family of apolipoproteins associated with high-density lipoprotein in plasma while apolipoprotein C-II is responsible for the activation of lipoprotein lipase. Circulating levels of free fatty acids, triglycerides, and cholesterol increase during sepsis. High-density lipoproteins have been shown to bind bacterial cell wall components, suggesting a role for lipoproteins in immune response modulation [9,10]. When compared to CRP, SAA levels rise more rapidly, peak on day 3 of sepsis, and return to baseline levels after four days [8].

Ng et al. in 2010 did a study using proteomic analysis investigating biomarkers in the context of diagnosis of LOS and necrotizing enterocolitis [11]. The study concluded that SAA and Apolipoprotein C-II were the most promising markers. Their plasma concentrations can be determined through immunoassay techniques and therefore can be incorporated into diagnostic tests in clinical practice. In this study, the “des-arginine” variant of SAA was used, which was the first time that this variant has been investigated in terms of LOS. It is also the first to investigate Apolipoprotein C-II in terms of neonatal rather than adult sepsis.

While the results of the study were not conclusive in showing the exact superiority of SAA, the researchers were able to develop a diagnostic equation, i.e., an ApoSAA score, which was used to score and stratify the infants by the risk of sepsis. It was also used to develop clinical guidelines for the initiation and discontinuation of antibiotics for each category of patients. 

4. Presepsin 

Presepsin is a soluble CD14 subtype that is released from the surface of immune cells due to stimulation by pathogens. Soluble CD14 is secreted by hepatocytes, suggesting it may be useful as an acute-phase reactant [12]. Notably, this marker has been shown to be a reliable diagnostic and prognostic marker of adult sepsis [12-14]. 

Poggi et al. performed a prospective study that involved 40 neonates, investigating the role of presepsin compared to CRP and PCT. Overall, the presepsin levels were higher in the LOS group compared to the control group and remained higher throughout the study period [14]. Presepsin demonstrated pooled sensitivity of 94% and specificity of 100%. 

Topcuoglu et al. carried out a prospective study involving 82 premature infants. The levels of presepsin, CRP, and PCT were measured on days 0, 3, and 7. At initial measurement, the presepsin levels in the LOS group were significantly higher than those in the control group. A presepsin value of 800.5 pg/mL was established as a cut-off value, with 67% sensitivity and 100% specificity [15]. The positive and negative predictive values were 100% and 74% respectively. Additionally, the presepsin levels showed a gradual decrease during and post antimicrobial therapy. 

Similar to the findings in the Poggi et al. study, the presepsin levels showed a decrease as early as day 1 post-treatment, whereas PCT and CRP levels did not differ from their baseline levels [14].

In 2019, Değirmencioğlu et al. investigated presepsin, comparing it to fetuin-A dyad. While most of the studies on fetuin fetuin-A have been done on animals, the authors felt it important to compare it to presepsin because it showed a valid connection to sepsis -- its levels declining in early sepsis and then increasing at 72 hrs of inflammation [16]. It is postulated that fetuin-A plays a role in the immune system by inhibiting the release of late mediators of inflammation. 

This study involved 55 preterm infants. Initial serum levels of presepsin, fetuin-A, CRP, and IL-6 were all higher in the LOS group compared to the control group. In contrast, fetuin-A levels were similar in both groups, suggesting it was not useful as a diagnostic marker. Overall, using a cut-off value of 823 ng/ml, the presepsin was found to have 88.9% sensitivity and 88.9% specificity [16]. 

5. Neutrophil CD64

CD64 (Fc-gamma receptor) is a high-affinity receptor, expressed on the surface of neutrophil granulocytes during bacterial infection. When a foreign antigen is recognized, opsonins such as immunoglobulin G (IgG) or complement components bind the antigen and form an IgG-antibody complex. The Fc fragment of the IgG molecule binds to specific neutrophil cell surface receptors such as Fc-gamma receptors and results in initiation of phagocytosis, degranulation, and antibody-dependent cellular cytotoxicity [17].

The role of CD64 in neonatal sepsis has been investigated in the past with promising results. A calculation of a CD64 index has been postulated to reduce bias from manual methods. However, very few studies have investigated this index in terms of LOS specifically. Increased expression of CD64 can be detected within one to six hours of bacterial invasion, and the levels remain elevated for up to 24 hours [3].

In a 2012 study by Mazzucchelli et al., CD64 was studied and compared to soluble TREM-1 [18]. Fifty four neonates were enrolled. Overall, CD64 levels were shown as increased significantly in the septic group versus the control group. At a cut-off value of 2.86, CD64 showed sensitivity of 87.52% and specificity 97.1% [18]. On the other hand, soluble TREM-1 did not show any differences in the septic group versus the control group. Therefore, it will not be discussed in this paper.

Later on, in 2015, Kipfmueller et al. investigated the potential of CD64 as a LOS marker. Notably, patients with EOS were not excluded from this study. Overall, 50 infants were enrolled [17]. It was found that CD64 index was elevated two days before sepsis was diagnosed. Overall, CD64 index was increased significantly in the sepsis group compared to the other groups [17]. A cut-off value of 1.86 was used, but the results were inconclusive due to the small sample size, and no actual values of sensitivity or specificity were calculated.

6. Endocan 

Endocan is a proteoglycan secreted by endothelial cells and is postulated to play a role in the pathogenesis of sepsis. An increase in its expression leads to endothelial activation and neovascularization, which are prominent pathophysiological changes associated with inflammation [19]. This is a novel biomarker that is being studied in terms of LOS. 

In a study by Buyuktiryaki et al., CRP, IL-6, and endocan levels were measured in a total of 102 preterm infants. Overall, while all three biomarkers showed “good performance” in discriminating sepsis and healthy controls, the area under the curve (AUC) values in the proven sepsis groups showed more of a significant value for endocan. Serial measurements went on to show that there was no significant difference in CRP and IL-6 levels between the proven and suspected sepsis groups while the endocan levels were significantly higher [19]. Overall, it was found that endocan showed a specificity of 94% and sensitivity of 94.2% [20] (Table 1).

**Table 1:** Summary of biomarkers discussed, their best-reported sensitivity and specificity values, and their corresponding timing in inflammatory response PCT, procalcitonin; CRP, C-reactive protein.

Biomarker	Sensitivity	Specificity	Timing in inflammatory response
PCT	88% [4]	72% [4]	Rises within 6 h of infectious insult, peaks at 18-24 h, and remains elevated for 48 h
CRP	89% [10]	>90% [10]	Rises within 12-24 h post-infectious insult and peaks at 48 h
Serum Amyloid A	Not reported	Not reported	Levels peak at 72 h and stay elevated until 96 h
Presepsin	94% [14]	100% [14]	Rises between 2-12 h after onset of infection and decreases as early as day 1 of treatment
CD64 index	87% [18]	97% [18]	Rises within 1-6 h post-infectious insult and can stay elevated up to 24 h
Endocan	94% [20]	94% [20]	Not reported

Discussion

LOS remains a major cause of mortality and morbidity worldwide [2]. An adequate blood test typically requires at least 1 ml of blood sample, and it is well documented that obtaining a sufficient quantity of blood from an ill neonate is difficult, and therefore false-negative results are common [20].

A consensus for a suitable diagnostic biomarker for LOS would be pivotal in preventing mortality and over usage of antimicrobials. There is currently no diagnostic tool for detecting LOS with 100% sensitivity, specificity, positive predictive value (PPV), and negative predictive value (NPV) [18]. The aim of this review was to focus on the studies that have been published investigating potential diagnostic biomarkers for LOS between the years 2010 and 2020. The goal is to highlight the strengths of each marker as a potential “gold standard” and discuss possible future studies. 

The work by Bustos et al. showed that PCT has more statistically significant differences when compared to CRP [4]. The authors highlight the PCT’s prognostic and therapeutic monitoring potential. 

In EOS studies, PCT-guided therapy has led to a significant reduction of time for antibiotic therapy. It is recommended that the same be studied for LOS. PCT has also been shown to have early detection post-infection compared to CRP. A strength of this study was that the PCT levels were measured post 72 hours of life, thus obviating any potential overlap with EOS. It is also worth noting that in septic adults, PCT has been used to monitor the progression of septic syndrome, evaluate treatment response, and estimate prognosis [21,22]. An important caveat to acknowledge is that PCT is triggered by other factors such as viral infections, perinatal trauma, or surgery. The ideal cut-off point for PCT is also still debated. A major limitation of the study is that only a small sample of neonates in a single center was investigated. Additionally, the assay used to measure PCT was susceptible to low sensitivity, and therefore moderate increases in PCT could have been missed.

The strength of the CRP study by Beltempo et al. was that it was performed over a longer period of time and offered serial measurements at three points in time. The consensus is that CRP is a traditional marker that shows limited use in the diagnosis of LOS. It is recommended that future research focuses on other more viable biomarkers that are elevated more quickly, in response to infection [3]. The work by Beltempo et al. showed that combining CRP with CBC offered higher sensitivity than CRP alone.

The literature on SAA and Apolipoprotein C-II indicates that they are more sensitive indicators than CRP. The extraordinarily high sensitivity and NPVnegative predictive value of the ApoSAA score proposed by Ng et al. make this screening strategy an appealing clinical option [20]. However, the generalizability of this tool to wider patient populations remains unknown. 

However, this study is the first and only study to focus on this marker; therefore, this is a significant limitation. As cited by the authors, the levels of SAA may also be affected by nutritional status and hepatic function. This study was also not specific for LOS. 

Presepsin studies show its promising prognostic and diagnostic potential. Several studies on adult sepsis have shown its diagnostic superiority compared to PCT [22]. One study reviewed in this literature showed that the presepsin levels decrease within 24 hours of treatment initiation compared to PCT and CRP. This suggests monitoring potential.

A strength of this study and the study done by Değirmencioğlu et al. was that serial measurements of presepsin were taken and compared over different days. 

While traditional biomarkers such as CRP and PCT are useful for excluding infection because they have high negative predictive accuracies, it is evident that they have relatively low positive predictive values [15]. The higher positive predictive values of presepsin suggest that it may be useful for LOS diagnosis. However, the presepsin levels of the patients who died from sepsis were not significantly different from the survivors. Therefore, one can infer that presepsin might not be as suitable for prognosis as hypothesized. 

While the study by Topcuoglu et al. showed the promising possibility for presepsin as a reliable biomarker, it was unable to demonstrate its efficacy for the detection of disease severity. The main limitation of this study was that they could not calculate AUC, sensitivity, and specificity for CRP and PCT in order to compare these markers with presepsin [15].

The study by Poggi et al. observed that presepsin may be useful as a treatment monitoring marker, even when used alone [14]. The 2019 study by Değirmencioğlu confirms presepsin as a seminal biomarker in diagnosis and monitoring of treatment response in LOS neonates. One weakness of this study is that only preterm infants with culture-proven sepsis were included but could have been compared to other groups. Overall, the three studies on presepsin used different cut-off values, so it is necessary to reach a consensus for clinically relevant use. 

The unambiguous conclusion from the CD64 studies is that it can be detected before the clinical symptoms of sepsis are detected [20]. This unique finding could aid earlier treatment. Additionally, CD64 can be measured by flow cytometry, which is a relatively rapid assay. One unanticipated finding was that an elevated CD64 index was detected in several infants in the control group. These episodes were described as asymptomatic inflammation. While they can be attributed to possible maternal inflammation or the presence of mechanical ventilation, this should still be noted [16]. A strong limitation is that one of the studies lacked a control group [17]. Furthermore, the cut-off of 1.86 does not seem to be appropriate in the first week of life, considering that more than 50% of infants in the control group had values >1.86 until day 7. 

In all the CD64 studies, the common limitation is a small sample size. Additionally, there is no consensus on CD64 cut-off values used across the studies. Overall, it seems that due to the relatively low specificity of CD64, being even lower than that of CRP, its diagnostic/screening power for LOS is arguable. There might be a potential for CD64 if used in combination with CRP as this has been shown in previous EOS studies. 

The endocan study showed that its levels decline much slower in sepsis cases compared to previous serum markers [19]. This could possibly allow a wider window of detection. Surprisingly, within this specific paper, when endocan was compared to CRP, it showed a relatively lower specificity/sensitivity. However, if we compare across all studies reviewed, endocan has a superior potential over CRP in terms of sensitivity. Like PCT, endocan has already been considered as a prognostic factor for adult sepsis, so further work should be done to investigate this for neonatal LOS. This study also indicated a potential for therapeutic monitoring. It is postulated that endocan might be more useful when used in combination with CRP/IL-6 [18]. A key limitation of this study was that only a small subgroup of neonates was studied in a single center. Furthermore, although patients with diseases that could influence endocan levels were excluded, it is still not yet fully understood which factors in preterm infants could affect endocan levels. 

As a possible suggestion for future studies, more novel evidence shows that there might be a pertinent role for biophysical markers, such as RALIS, when used in conjunction with serum biomarkers. This is a computerized algorithm device that requires the clinician to input various data including heart rate, respiratory rate, core body temperature, body weight, number of desaturation events, and number of bradycardic events [3]. This can be used to make a more accurate diagnosis. 

The strength of this review is that it achieved the aim of identifying the diagnostic biomarkers of LOS that have been investigated thus far and highlighted the gaps/inconsistencies that exist. In relation to the individual biomarkers, it revealed which ones demonstrate relatively superior predictor abilities. Collectively, majority of the studies compared the biomarkers to CRP and PCT, thus enabling a valid comparison to be made across all studies.

It has been reported that an ideal biomarker for neonatal sepsis should have a sensitivity of 100%, specificity > 85%, PPV > 85%, and NPV of 100 [19]. This review highlights that more investigations are needed to find this ideal biomarker. One can conclude that no single biomarker is faultless, considering the variable results discussed. Rather, the review has shown that combinations of biomarkers as well as incorporating newer algorithms could be used to identify and risk-stratify appropriately.

The limitation of this review was the timeframe over which studies were selected. Only studies from 2010 to the present were reviewed. Additionally, there were no hand-selected studies reviewed with all studies being obtained from databases. Furthermore, due to the heterogeneity of the studies, it is clear that there needs to be a consensus on cut-off values. Overall, larger studies in multicenters including infants of varying gestational ages are also necessary (Table 2).

**Table 2:** Summary of strengths and limitations of the biomarkers discussed within the review PCT, procalcitonin; CRP, C-reactive protein; LOS, late-onset sepsis.

Biomarker	Strengths	Limitations
PCT	- Prognostic and therapeutic potential	- Can be triggered by non-infectious states such as perinatal trauma
	- Shows early detection post-infection onset (superior to CRP)	- No consensus in cut-off values across studies
	- Ultrasensitive methods for detection are available	- PCT-guided therapy has not yet been developed for neonates < 32 weeks
CRP	- Can give serial measurements	- Limited role as a sole marker for LOS diagnosis
	- Potential for higher diagnostic accuracy when combined with CBC	- Rises relatively slower post-onset of infection
Serum Amyloid A	- Shows rapid elevation post-infection onset	- Minimal information available on the marker in terms of LOS specifically (one study reviewed)
	- Prognostic potential	- Levels might be confounded by nutritional status and hepatic function
	- Easily measured	
	- ApoSAA score can be applied for prompt screening and proper treatment allocation	
Presepsin	- Prognostic potential	- Questionable prognostic potential
	- Levels decrease rapidly in response to treatment, thus can be used to monitor the efficacy of treatments	- Cut-off values lack consensus
	- Allows for serial measurements	
	- Superior sensitivity, specificity, and high positive predictive values	
CD64 index	- Can be detected relatively early, even prior to the development of clinical symptoms	- Levels noted to rise due to non-infectious states
	- Rapid assay measurement possible	- No consensus on cut-off values
	- Better potential when used in combination with CRP	- Lower specificity than CRP
Endocan	- Stays elevated longer	- Novel biomarker therefore more studies needed
	- Superior sensitivity and specificity, comparable to presepsin	
	- Prognostic potential	
	- Therapeutic monitoring potential	
	- Can be used in combination with CRP/IL-6 to improve accuracy	

## Conclusions

Blood culture remains the diagnostic gold standard for LOS. There is a necessity to develop the ideal marker to aid diagnosis and screen treatment monitoring. Trends seem to be moving away from traditional markers such as CRP and PCT. SAA and CD64 show promising potential, showing strengths higher than CRP. Presepsin and endocan appear to have superior overall potential, useful for diagnosis, prognosis, and treatment response. Work on the more novel biomarkers compared to more traditional ones is limited. Nevertheless, the high diagnostic potential of these novel markers should prompt further work. There is still no single biomarker identified for LOS diagnosis, and the small number of studies reviewed identifies gaps in current knowledge and ascertains the necessity for further investigation. Consensus on an ideal biomarker will be vital to aid proper diagnosis and adequate treatment, which should reduce mortality levels associated with neonatal LOS. 

While imperative not to forget the current value of clinical examination in LOS diagnosis, it is necessary to find the ideal biomarker that can be used in conjunction with clinical vital signs monitoring tools. This would be akin to the neonatal early-onset sepsis calculator (NEOSC). This is a tool aimed at aiding clinical decisions, based on using risk factors such as gestational age, highest maternal antepartum temperature, maternal group B Strep infection (GBS) status, incidence of EOS, duration of rupture of membranes (ROM), and type of intrapartum antibiotics used. It helps to calculate the risk of EOS for infants born >34 weeks of gestation. With this, clinicians can decide whether to start treatment and choose duration, etc. This tool has helped reduce investigation time and unnecessary antibiotic use considerably, with no difference in subsequent sepsis rates. Further work is therefore needed to find an equivalent tool in the context of LOS.
